# A Homolog of the Histidine Kinase RetS Controls the Synthesis of Alginates, PHB, Alkylresorcinols, and Motility in *Azotobacter vinelandii*

**DOI:** 10.1007/s00284-024-03835-1

**Published:** 2024-08-17

**Authors:** Araceli Rosales-Cruz, Jimena Reyes-Nicolau, Eduardo Minto-González, Alan Meneses-Carbajal, Claudia Mondragón-Albarrán, Liliana López-Pliego, Miguel Castañeda

**Affiliations:** Centro de Investigaciones en Ciencias Microbiológicas, Instituto de Ciencias, Benemérita Universidad Autónoma de Puebla, IC-11 Ciudad Universitaria Puebla, Apdo. Postal 1622, C. P. 72000 Puebla, Pue Mexico

## Abstract

The two-component system GacS/A and the posttranscriptional control system Rsm constitute a genetic regulation pathway in Gammaproteobacteria; in some species of *Pseudomonas*, this pathway is part of a multikinase network (MKN) that regulates the activity of the Rsm system. In this network, the activity of GacS is controlled by other kinases. One of the most studied MKNs is the MKN-GacS of *Pseudomonas aeruginosa*, where GacS is controlled by the kinases RetS and LadS; RetS decreases the kinase activity of GacS, whereas LadS stimulates the activity of the central kinase GacS. Outside of the *Pseudomonas* genus, the network has been studied only in *Azotobacter vinelandii*. In this work, we report the study of the RetS kinase of *A. vinelandii*; as expected, the phenotypes affected in *gacS* mutants, such as production of alginates, polyhydroxybutyrate, and alkylresorcinols and swimming motility, were also affected in *retS* mutants. Interestingly, our data indicated that RetS in *A. vinelandii* acts as a positive regulator of GacA activity. Consistent with this finding, mutation in *retS* also negatively affected the expression of small regulatory RNAs belonging to the Rsm family. We also confirmed the interaction of RetS with GacS, as well as with the phosphotransfer protein HptB.

**Supplementary Information:**

The online version contains supplementary material available at 10.1007/s00284-024-03835-1.

## Introduction

In bacteria, signal transduction is generally performed by proteins belonging to signaling systems known as two-component systems (TCSs). TCSs are composed of a receptor protein (histidine kinase, HK) that, upon receiving a signal, autophosphorylates a histidine residue in the transmitter domain (H1) and activates, through transphosphorylation, the second element of the system known as the response regulator (RR). RR is phosphorylated on an aspartate residue of its receptor domain, also known as REC or D2 [1]. The HK-RR regulatory pair paradigm has recently been questioned because of the existence of systems with two or more HKs that control the phosphorylation status of an RR but where only one HK phosphorylates the RR. The accessory HK (or HK) is proposed to stimulate or block the kinase activity of the main HK. These signal transduction systems are known as multikinase networks (MKNs), and their study has become a novel area of research in physiology and signaling in prokaryotes [2]. GacS/A is a common TCS in Gammaproteobacteria and is considered a global regulatory system of secondary metabolism [3]. The regulation exercised by GacS/A uses the posttranscriptional control system Rsm (Csr) as an intermediary, which consists of one or more proteins and two or more small regulatory RNAs (sRNAs) [3, 4]. The protein(s) of the Rsm (Csr) system, encoded by the *rsmA* (*csrA*) gene, bind to its mRNA targets and, in most cases, block their translation, and promote their degradation, although they can act via other mechanisms [5]. The sRNAs-Rsm counteracts the functions of RsmA, and GacA positively controls its transcription [4]. The TCS GacS/A in *Pseudomonas aeruginosa* is related to other HKs. It forms a MKN in which GacA activity is negatively controlled by RetS and positively controlled by the HK LadS [2, 6]. Thus, GacS, RetS, and LadS constitute the core of the GacS-MKN that controls the activation of GacA, which in turn regulates the transcription of *rsmZ* and *rsmY*, the genes that encode the sRNAs of the Rsm system [2, 7]. Another HK, PA1611, has been reported to block the activity of RetS on GacS [8]. GacS is an unorthodox HK. In addition to the transmitter or H1 domain, this type of kinase has two extra phosphorylation domains: a receiver domain (D1) and a domain-designated H2 or Hpt. Recently, a new pseudoreceptor domain (referred to as the ND) was identified in GacS. This domain is situated between the transmitter and receiver domains and is essential for autokinase activity [9]. In these kinases, phosphate is transferred sequentially from the H1 domain to D1 and from D1 to H2, which transfers the phosphate to the D2 domain of the RR [3]. RetS, LadS, and PA1611 are hybrid HKs; these kinases are similar to unorthodox HKs but do not have an H2 (Hpt) domain [8, 10]. Thus, only GacS can phosphorylate GacA, while the hybrid HKs of this MKN stimulate GacS phosphorylation or promote its dephosphorylation [2, 6, 8].

On the other hand, PA1611 phosphorylates a soluble protein that carries an Hpt domain-designated HptB, which, in turn, indirectly activates the expression of the sRNA RsmY. In addition to PA1611, the kinases ErcS’ and SagS can also phosphorylate HptB, and together with PA1611, form part of the HptB branch of GacS-MKN [8, 10]. In *Pseudomonas fluorescens*, *Pseudomonas protegens*, *Pseudomonas putida,* and *Pseudomonas syringae*, there are orthologs of these kinases that constitute the core of the GacS-MKN, but their roles in GacS-MKN have not been characterized [11–13]. Outside of the genus *Pseudomonas*, only in *Azotobacter vinelandii* has this MKN been studied [14].

*A. vinelandii* is a nitrogen-fixing soil bacterium that forms cysts resistant to desiccation and produces three metabolites of biotechnological interest: alginates, polyhydroxybutyrate (PHB), and alkylresorcinols (ARs) [15–17]. Alginates are a family of polymers composed of monomers of guluronic acid and mannuronic acid joined by links β (1–4). In industry, alginates are used as additives for viscosifying, stabilizing, emulsifying, and gelling aqueous solutions [18]. PHB is a polyester consisting of β-hydroxybutyrate monomers belonging to the polyhydroxyalkanoate (PHA) family. PHAs are generally synthesized and stored as a source of carbon and energy. PHAs are interesting compounds since they can form plastics with properties similar to those of polypropylene and polyethylene with the advantage of being biodegradable [19]. ARs are long-chain phenolic lipids. The ARs synthesized by *A. vinelandii* are 5-n-eneicosylresorcinol, 5-n-tricosylresorcinol, and their galactosidase derivatives, which, together with alkylpyrones, replace the phospholipids in the membrane during encystment and are part of the cyst covering [17]. ARs have potential uses as immunomodulators and anticancer and antimicrobial agents [20]. GacS/A regulates alginates, polyhydroxybutyrate (PHB), and ARs through the posttranscriptional control system Rsm, which specifically controls the transcription of the sRNAs RsmZ1, Z2, Z3, Z4, Z5, Z6, Z7, Z8, and RsmY [21–25]. Previously, in *A. vinelandii*, the presence of a *retS* homolog was reported and shown to interact with GacS, although it has not been thoroughly characterized [14]. Interestingly, this bacterium does not have a *ladS* homolog but possesses another HK (designated HrgS) functionally related to GacS; thus, in *A. vinelandii*, unlike that reported in *P. aeruginosa*, the core of GacS-MKN could be integrated by GacS, HrgS, and RetS [14]. This work reports the phenotypic characterization of the *A. vinelandii retS* homolog and its probable function in the GacS-MKN.

## Materials and Methods

### Microbiological Procedures

The bacterial strains used are listed in Table S1. *A. vinelandii* strains were grown at 30 °C in Burk’s nitrogen-free medium with salts [26] supplemented with 20 g/L sucrose (BS). *Escherichia coli* strain DH5-α was grown on Luria–Bertani (LB) medium at 37 °C. The antibiotic concentrations used (in μg/mL) for *A. vinelandii* and *E. coli* were as follows: tetracycline (Tc), 40 and 20; kanamycin (Km), 4 and 20; gentamicin (Gm), 1.5 and 10; streptomycin (Sm) 2 and 20; ampicillin (Ap), 0 and 100; nalidixic acid (Nal), 10 and 10. *A. vinelandii* transformation and conjugation were carried out as previously described [27].

### Nucleic Acid Procedures

DNA isolation and cloning procedures were carried out as described previously [28]. DreamTaq polymerase and Fusion High FidelityDNA polymerase (Thermo Fisher Scientific) were used for PCR amplification. The *A. vinelandii* DJ [29] genome sequence was used to design the oligonucleotides for PCR amplification.

## Generation of *A. vinelandii retS* Mutants

A 1581-bp DNA fragment containing *retS* was amplified by PCR from *A. vinelandii* E [30] chromosomal DNA with the primers FRetSZ-BH1 and RRetS-S1. The oligonucleotides used were designed from the DJ strain genome sequence (Table S1). This fragment was subsequently cloned and inserted into the pGEMT-Easy vector, and the resulting plasmid was designated pGEM*retS*1.5. This plasmid was used to determine the nucleotide sequence of the *retS locus* of strain E, which showed 100% identity with the corresponding sequences of the DJ strain. To construct the *A. vinelandii retS* mutant, the plasmid pGEM*retS*1.5 was cleaved at the single *Cla*I site located within the sequence corresponding to the transmitter domain of this HK. A 1200-bp *Cla*I fragment carrying a Km resistance cassette obtained from pBSL98 [31] was subsequently ligated into this plasmid; the resulting plasmids were designated pGEM*retS*::KmNP and pGEM*retS*::KmP. In the first plasmid, the resistance cassette was inserted in the same sense orientation as that of the *retS* gene; in the second plasmid, the Km cassette was inserted in the opposite sense orientation to that of the *retS* gene. In *A. vinelandii*, the insertion of resistance cassettes into genes with the same orientation as the direction of transcription produces nonpolar mutations, which allow transcription of the downstream genes in the same operon; otherwise, insertions in the opposite sense generate polar mutations [32]. Later, competent cells of the wild-type strain E were transformed with the plasmids pGEM*retS::*KmNP and pGEM*retS*::KmP that were previously linearized with *Sca*I to ensure allelic exchange by double reciprocal recombination events. Km-resistant transformants were selected, and the corresponding mutations and the absence of wild-type *retS* alleles were confirmed by PCR analysis and subsequent sequencing (data not shown). The resulting mutants were designated E*retS*NP and E*retS*P.

Plasmid pGEM*retS*::KmNP was used as described above to generate mutations in strains that carried a transcriptional fusion of the genes *rsmZ1* (EP*rsmZ1*-*gusA*), *rsmZ2* (EP*rsmZ2*-*gusA*), *rsmZ6* (EP*rsmZ6*-*gusA*), and *rsmY* (EP*rsmY*-*gusA*) [24, 25]. The resulting strains were designated EP*rsmZ1*-*gusAretS*, EP*rsmZ2*-*gusAretS*, EP*rsmZ6*-*gusAretS,* and EP*rsmY*-*gusAretS*.

### Construction of *retS gacS* Double Mutants

To generate the double mutant E*retSgacS*, the corresponding single mutant *retS* was conjugated with the plasmid pSUP*gacS*::Sm, which carried a nonpolar mutation generated by the insertion of a streptomycin cassette in *gacS*. pSUP*gacS*::Sm was constructed by subcloning the insert of pMC7 [33] into pSUP202 [34]. Afterward, Sm-resistant transconjugants were selected, and the success of the double recombination was verified by sequencing and PCR analysis with the primers WTS2D and WTS2R (Table S1) (data not shown).

### Complementation Analysis of the EretS Mutant

To carry out genetic complementation analysis of the mutant E*retS,* a 3069-bp DNA fragment was amplified with the primers ER1RR_RetS_Fw and RRetS-S1 (Table S1) and subsequently cloned and inserted into pGEMT-Easy, generating the plasmid pGEM*retS*wt. The sequence inserted into pGEM*retS*wt was subcloned and inserted into pUMATc [35]; pUMATc is an integrative suicide vector that promotes the integration of the cloned DNA into the *melA* locus of the *A. vinelandii* chromosome. Transformants resistant to Tc were isolated and confirmed by PCR analysis to carry the *retS locus* inserted into the *melA* gene. PCR was performed with the ER1RR_RetS_Fw and Hsp70_RTIRv primers (Table S1) (data not shown). The recombinant strain was designated E*retS*/*melA::retS*.

### Two-Hybrid LexA Assay

This assay, which was carried out in *E. coli*, allows the interaction between two proteins to be studied. LexA is a repressor that acts as a dimer. An interaction domain is required to form the dimer, which is removed and replaced by the domains of the proteins to be tested. If this occurs, a dimer is formed that represses the expression of *lacZ* located on the chromosome of the reporter strain of the system. To carry out the LexA two-hybrid assay [36] between GacS and RetS, the plasmids pSR659GacS and pSR658RetS were used. Similarly, these plasmids, along with pSR658RetS and pSR659HptB, were used to determine the RetS and HptB interaction. The plasmid pSR659HptB was constructed for this study by cloning a PCR fragment corresponding to the coding region of *hptB*; this fragment was amplified with the primers HptBTH SacIFw and HptBTHKpnIRv (Table S1). The plasmid pSR659GacS combined with pSR658RetS was cotransformed into the *E. coli* strain SU202, and the effects of the protein interactions were visualized on MacConkey-lactose indicator plates. A similar assay was performed for RetS and HptB with the plasmids pSR658RetS and pSR559HptB.

### Motility Assays

To perform the motility assays, the bacterial strains were grown on BS medium at 30 °C until they reached the exponential phase (24 h). Samples of the cells (1X10^5^ CFU) were then transferred to BS plates containing 0.15% agar for swimming tests. These plates were incubated at 30 °C for 24 h.

### Analytical Methods

Protein content was determined by the Lowry method [37]. Alginate production was determined as previously described [38]. ARs synthesis was measured as reported previously [39]. The PHB content of the bacteria was assayed by the method of Law and Slepecky [40], and β-galactosidase activities were determined as previously reported [41]. All the measurements were performed in triplicate. Glucuronidase activity was measured as previously reported [42]. One U corresponds to 1 nmol of *O*-nitrophenyl-β-d-glucuronide hydrolyzed per min per µg of protein.

The sequence accession numbers of the *A. vinelandii* strains used in this work were as follows: DJ strain, GenBank CP001157; E (AEIV) strain, GenBank CP092752.

## Results

### Search for Putative *retS* Homologs in *A. vinelandii*

We initiated our investigation of the *A. vinelandii retS* gene with Avin_6870, a putative gene annotated as a *retS* homolog in the genome sequence of the DJ strain. The DJ strain is a nonmucoid type strain of *A. vinelandii* [29]. This gene has a 48% identity with *retS* of *P. aeruginosa*, while the protein it encodes has a 57% identity with its *P. aeruginosa* counterpart. The prediction of the protein domains revealed the characteristic architecture of RetS homologs, which feature two receiver domains and an unusual 7TMR-DISMED2 input domain in addition to the transmitter domain [6]. The transmitter domain (H1) of the RetS homolog of *A. vinelandii* has a high identity (69%) with its *P. aeruginosa* counterpart (Fig. S4). These unique features of RetS homologs are crucial for understanding the protein’s function and potential implications (Fig. S1).

Previously, a 3.5 kb fragment containing *retS* and its regulatory region from the wild-type mucoid strain E (also named AEIV) was amplified and sequenced. We decided to work with this strain because it produces alginate, and much of our work has focused on studying the production of this polymer. The sequence was found to be practically identical to its counterpart in the DJ strain, with a remarkable 99% identity. Recently, the genome of strain E (AEIV) was released in the GenBank database. The sequence of the *retS* locus reported in GenBank was 100% identical to that obtained in this work. In *A. vinelandii*, a gene that encodes a heat shock protein belonging to the Hsp70 family is located 100 bp downstream of *retS*. Due to the proximity between these genes, mutations in *retS* could affect *hsp70*.

### RetS Regulates the Synthesis of Alginates in Strain E

By allelic exchange and the use of previously described mutagenic plasmids (see “Materials and Methods” section for details), we generated E*retS*-polar and E*retS*-nonpolar mutants (E*retS*P and E*retS*NP, respectively). To test the polar and nonpolar nature of the insertions, we performed RT-PCR amplification of a region downstream of the insertions (Fig. S2b); lines 3 and 4 correspond to the samples of the polar and nonpolar mutants, respectively. The absence of amplification in line 3 proved the polarity of the insertion in the E*retS*P mutant. The amplification shown in line 4 corresponds to the nonpolar mutant (E*retS*NP), which shows that the insertion did not affect the integrity of the *retS* mRNA, this is consistent with the published findings regarding these types of mutations [32]. Furthermore, to rule out a polarity effect on the expression of the *hsp70* gene, we performed a transcriptional analysis of *hsp70* by RT-PCR. The results are shown in Fig. S2c, which shows that the expression of *hsp70* was not affected in the E*retS*P (line 3) and E*retSN*P (line 4) mutants.

**Fig. 1:**
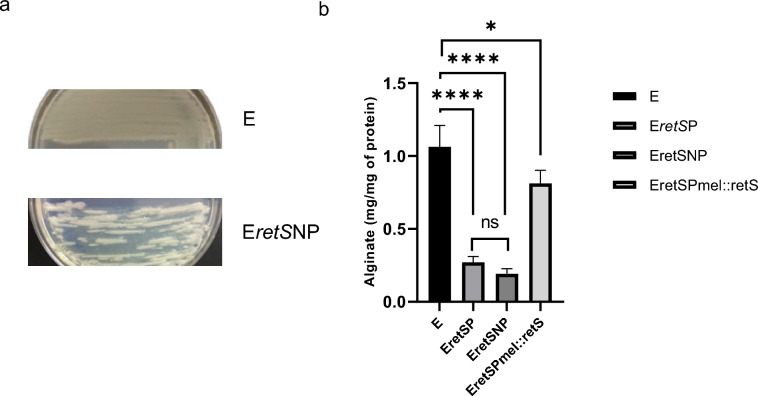
Regulatory effect of AvAEIV_000681 (*retS*) on alginate synthesis. **a** Mucoid phenotypes of wild-type strain E and its derivate with an AvAEIV_000681 (*retS*) mutation. **b** Alginate production in *A. vinelandii retS* mutants. All the measurements were done in cells grown for 48 h in Burk’s minimal media with sucrose. The bars represent the statistical media of three measurements and their standard deviation. Significant differences were analyzed by Anova test. Statistical significance is indicated. *P* < 0.1234; ns (not significant), *P < 0.03232; **P < 0.021; ***P < 0.0002; ****P < 0.0001

The E*retS* mutants presented a diminished mucoid phenotype; another relevant phenotypic characteristic was irregular and discontinuous growth on agar (Fig. 1a). In *A. vinelandii*, the mucoid phenotype is directly related to alginate synthesis; this finding was corroborated by the quantification of alginate production in the E*retS* mutants [43]. Polar and nonpolar mutants had similar effects on alginate production (Fig. 1b), on the other hand, introducing the wild-type allele of *retS* into the E*retS*P mutant restored alginate synthesis. The polarity analysis previously described, together with the complementation of the E*retS*P mutant with the wild-type gene (Fig. 1b), revealed that the effect observed in the synthesis of alginates was only due to the absence of *retS*. On the basis of these results, we decided to continue characterizing *retS* in the polar and nonpolar mutants.

### RetS Also Controls Other Phenotypes Related to HK GacS

In *Pseudomonas* species, RetS acts as a negative regulator; accordingly, the production of alginates in the E*retS* mutant should increase; however, Fig. 1b shows that the opposite was true. These data suggested that, in *A. vinelandii*, RetS acts as a positive regulator. To verify the regulatory character of RetS, we measured the production of other GacS-controlled metabolites in *A. vinelandii*, such as PHB and ARs [22, 23].

**Fig. 2:**
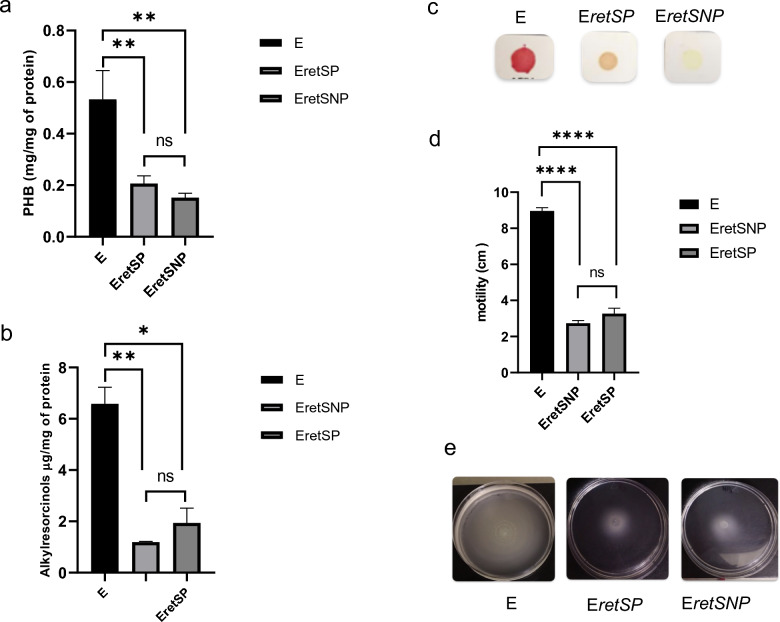
Effect of the *retS* mutations on ARs synthesis, PHB accumulation, and swimming motility. **a** PHB production in E WT strain and its mutant derivatives *retS*. PHB content was determined in cells grown for 48 h in PY liquid medium supplemented with 2% sucrose. **b** ARs synthesis in wild-type strain E and its mutant derivatives *retS*. The ARs measurements were done in cells grown for 120 h in Burk’s minimal media amended with *n*-butanol (0.2%) as a carbon source. In both graphs, the bars represent the statistical media of three measurements and their standard deviation. Significant differences were analyzed by ANOVA test. Statistical significance is indicated. *P* < 0.1234; ns (not significant), *P < 0.03232; **P < 0.021; ***P < 0.0002; ****P < 0.0001. **c** Colonies of *A. vinelandii* E (wild type), and its derivatives mutants *retS*, Fast Blue B stain makes the ARs production visible with a reddish color. The *A. vinelandii* strains grew on Burk media amended with 2% of Butanol (BBOH medium) for 120 h of incubation. **d** Quantitative motility assay of *A. vinelandii* wild-type strain E and its derivatives *retS* mutants. The cells were cultivated over BS plates amended with 0.15% agar to test swimming motility. The motility halo was visualized and measured after 24 h of incubation. The bars represent the statistical media of three measurements and their standard deviation. Significant differences were analyzed by Anova test. Statistical significance is indicated. *P* < 0.1234; ns (not significant), *P < 0.03232; **P < 0.021; ***P < 0.0002; ****P < 0.0001. **e** Swimming motility phenotypes of *A. vinelandii* wild-type strain E and its derivatives *retS* mutants

Our experiments also revealed a significant reduction in PHB synthesis as a consequence of the *retS* mutation. The data in Fig. 2a highlight the crucial role of RetS in this process.

The effect of the *retS* mutation on ARs synthesis was evident upon fast blue staining, as shown in Fig. 2c. The absence of *retS* hindered ARs synthesis, a finding that was subsequently confirmed quantitatively (Fig. 2b).

The GacS-Rsm pathway is also involved in controlling swimming motility in *A. vinelandii*. Therefore, we investigated whether mutation of *retS* could indirectly affect motility. The reduced motility in *retS* mutants (shown in Fig. 2d, e) strongly suggests that this histidine kinase, along with GacS, was involved in flagellum biosynthesis on the basis of the function reported for GacS in *A. vinelandii* [44].

### RetS is Involved in the Transcriptional Control of Genes Encoding sRNAs of the Rsm Family

The phenotypes related to GacS that were affected in the E*retS* mutant suggest that as in *Pseudomonas* spp., Rsm-sRNAs could be a regulatory target of RetS [6]. To prove this, the *retS* mutation was transferred into strains carrying *gusA* transcriptional fusions of genes encoding some of the *A. vinelandii* Rsm-sRNAs. *A. vinelandii* has eight sRNAs belonging to the Rsm family, with seven of the RsmZ (RsmZ1-7) subfamily and one of the RsmY subfamily [24, 25]. After ruling out the polarity mutation effect, the experiments were carried out only with strains derived from the E*retS*NP mutant because similar effects were observed for the E*retS*P and E*retS*NP mutants.

**Fig. 3:**
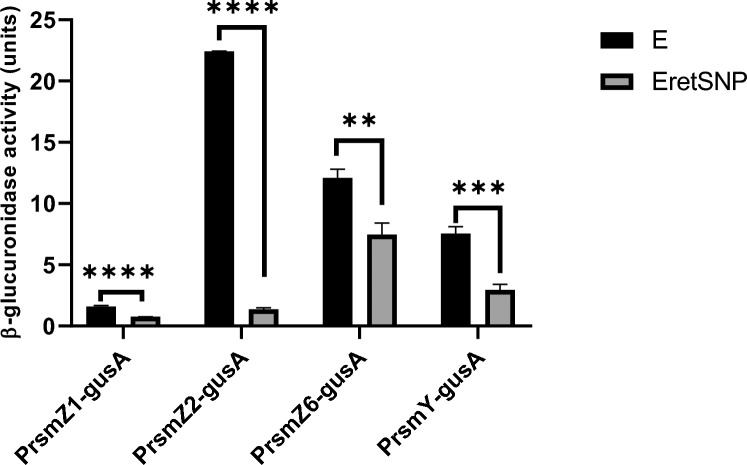
Effect of the *retS* mutation on the activity of the P*rsmZ1*-*gusA*, P*rsmZ2*-*gusA*, P*rsmZ6*-*gusA*, and P*rsmY*-*gusA* transcriptional fusions. Promotor activity of the transcriptional fusions P*rsmZ1*-*gusA*, P*rsmZ1*-*gusA*, and P*rsmZ1*-*gusA* in strains EP*rsmZ1*-*gusA*, EP*rsmZ1*-*gusA* and EP*rsmZ1*-*gusA*, and their derivative mutants *retS*. All the measurements were carried out in cells grown for 48 h in Burk’s minimal media with sucrose. The bars represent the statistical media of three measurements and their standard deviation. The bars represent the statistical media of three measurements and their standard deviation. Significant differences were analyzed by *t* test. Statistical significance is indicated. *P* < 0.1234; ns (not significant), *P < 0.03232; **P < 0.021; ***P < 0.0002; ****P < 0.0001

Figure 3 shows the transcription of four of the eight Rsm-sRNAs, *rsmZ1*, *rsmZ2*, *rsmZ6*, and *rsmY*; in all the cases, the *retS* mutation diminished the expression of the *rsm*-sRNAs genes.

### GacS is Required for the RetS Function

The HK GacS of *A. vinelandii* has a DHp subdomain and a HAMP domain that are highly conserved with their counterparts in GacS of *P. aeruginosa*. Both domains possess up to 70% identity (Fig. S3), suggesting that functional relationships similar to those of its *P. aeruginosa* homolog could be established.

**Fig. 4:**
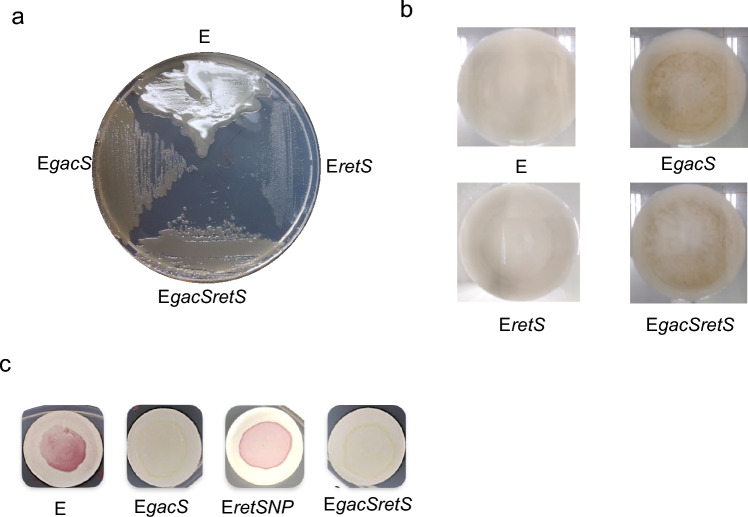
Phenotypes of single mutants E*retS* and E*gacS* and double mutant E*retSgacS*. **a** Mucoid phenotypes of the *A. vinelandii* strain E (wild type), E*gacS*, E*retS*, and the double mutant E*gacSretS* growth on Burks Sucrose (BS medium) semisolid medium at 30 °C for 48 h. **b** Flocculation phenotype of the strains E*gacS*, E*retS*, and the double mutant E*gacSretS* growth on BS liquid medium at 30 °C, 2000 rpm for 48 h. **c** Staining of ARs produced by *A. vinelandii* strains E (wild type) E*gacS*, E*retS*, and the double mutant E*gacSretS* growth on Burk Butanol (BBOH medium) for 120 h of incubation

The data presented, thus, far strongly suggest a functional relationship between GacS and AvAEIV_000681 (RetS). To test this hypothesis, we created a double mutant, E*gacSretS*, and conducted phenotypic hierarchy studies alongside the single mutants *retS* and *gacS*. While both the *retS* and *gacS* single mutants presented reduced alginate production, they presented different phenotypes. In semisolid media, the *gacS* mutant displayed the growth of colonies that were rough and dark (Fig. 4a), whereas in liquid media, it formed flocs (Fig. 4b). Conversely, *retS* mutants produced clear colonies in semisolid media and did not flocculate in liquid medium (Fig. 4a). In the E*gacSretS* double mutant, the *gacS* phenotype was dominant (Fig. 4a), confirming the genetic relationship between the two kinases. Additionally, the dominance of the *gacS* phenotype indicated the greater genetic hierarchy of *gacS* over *retS*.

Previously, a null ARs production phenotype was reported in *gacS* and *gacA* mutants [23], which contrasts with the partial phenotype of the E*retS*P mutant. Interestingly, the partial ARs production phenotype in the E*retS*P mutant became a null ARs production phenotype through mutation of the *gacS* gene. As in the previous case, in the *EretSgacS* double mutant, the phenotype of the *gacS* mutation prevailed (Fig. 4c).

### RetS Interacts with HptB

**Fig. 5:**
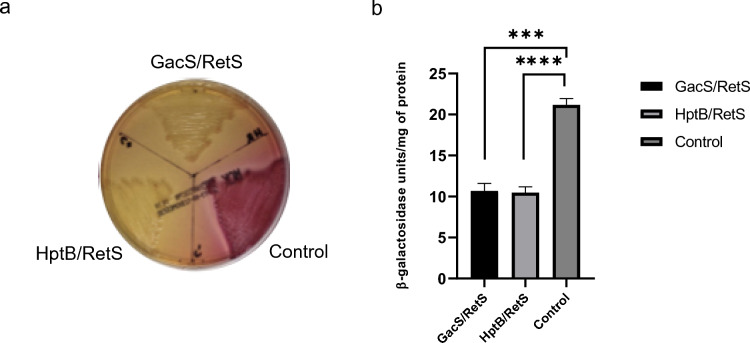
Determination of the interaction of RetS with GacS and HptB, established by LexA Two-hybrid assays. **a** Plate Two-Hybrid assay performed with RetS, GacS, and HptB. **b** Quantitative Two-Hybrid assay carried out with RetS, GacS, and HptB. The LexA dimerization domain was removed and replaced with GacS, RetS, and HptB proteins. Since LexA is an active repressor only as a dimer, dimerization of the tested proteins could allow chimeric LexA to bind to its operator site and repress transcription of the *lacZ* reporter gene, resulting in a lactose-negative phenotype of the *E. coli* reporter strain. The bars represent the statistical media of three measurements and their standard deviation. Significant differences were analyzed by *t* test. Statistical significance is indicated. *P* < 0.1234; ns (not significant), *P < 0.03232; **P < 0.021; ***P < 0.0002; ****P < 0.0001

In a previous study in *A. vinelandii*, the physical interaction between RetS and GacS was reported [14], whereas in *P. aeruginosa*, RetS has also been reported to interact with the HptB protein; it has been proposed that HptB can phosphorylate RetS [10]. To establish whether this also occurs in *A. vinelandii*, a LexA two-hybrid assay was carried out. To perform the assay, we cloned DNA fragments corresponding to the cytoplasmic domains of the *gacS* and *hptB* genes into LexA expression vectors. The absence of a color change in the MacConkey medium confirmed the interaction between RetS and HptB (Fig. 5a). The interaction assay between RetS and GacS was repeated as a positive control, confirming the interaction between RetS and GacS. Figure 5b shows the quantitative results of the assays.

## Discussion

The study of MKNs in bacterial signaling is an emerging area of research that has improved the understanding of how bacteria sense and respond to their environment [2]. The *P. aeruginosa* GacS network is one of the most studied MKNs; bioinformatic searches suggest the existence of this MKN in many bacteria of the *Pseudomonas* genus [8], but it has been partially characterized only in *Pseudomonas protegens* (formerly *Pseudomonas fluorescens*) [11] and *Pseudomonas syringae* [45]. GacS forms the core of the GacS-MKN as the central kinase, whereas RetS and LadS are its regulatory kinases [2]. In *P. aeruginosa*, LadS promotes alternative GacS phosphorylation, increasing GacA phosphorylation. To carry out GacS phosphorylation, LadS requires an REC domain [8]. Interestingly, in *P. syringae*, LadS also acts as a positive regulator; however, it does not contain the REC domain, suggesting a distinct regulatory mechanism [45].

In *P. aeruginosa,* RetS acts as a negative regulator, forming heterodimers with GacS. There are three mechanisms through which RetS blocks the function of GacS: inhibition of GacS autophosphorylation, removal of the phosphate from the phosphorylated GacS, and dephosphorylation of the REC domain of phosphorylated GacS [46]. Forming the GacS heterodimer requires the DHp subdomain (a subdomain of the transmitter domain) and an additional HAMP domain, which GacS possesses just above the transmitting domain. The DHp subdomains of both proteins interact with each other, and the GacS HAMP domain contacts the RetS transmitter domain in a region distinct from the DHp subdomain [9]. The high identity and domain conservation between *A. vinelandii* and *P. aeruginosa* RetS and GacS are in agreement with the interaction results. Interestingly, and counter to what was expected, the *retS* mutation diminished alginate production. In *A. vinelandii*, GacS positively controlled alginate production. Thus, mutations in *gacS* also decreased alginate synthesis. The regulatory effect of RetS on alginate synthesis was likely due to its interaction with GacS; therefore, a mutation in *retS* was expected to affect other GacS phenotypes. For that, we tested other phenotypes regulated by GacS, and the results were consistent with the positive regulatory character of RetS found for the synthesis of alginates, thus, ruling out the exceptional situation described above. The positive regulatory effect of RetS is not common; it has only been reported in the control of swimming, swarming, and surfactant production in the strain Pf5 of *P. protegens* [47]. Interestingly, in the same strain, the production of the antifungal compound 2,4-diacetyl phloroglucinol is negatively regulated by RetS [48]. This last case correlates with the regulatory mechanism originally described for RetS. Overall, previously reported data [47, 49] suggest the existence of alternative mechanisms through which RetS performs its regulatory functions.

The high similarity of GacS and RetS of *A. vinelandii* with those of its homologs from *P. aeruginosa* and the results obtained from the two-hybrid assay suggest that the formation of the GacS-RetS heterodimer, which would prevent the transphosphorylation of GacS, is highly probable. It is widely documented that GacS homologs are not phosphorylated by a cis mechanism [48]. Thus, the heterodimer turns off autokinase activity. The transmitter domain of RetS homologs has structural alterations that turn off its kinase activity; the high conservation of the primary and predicted tertiary structures of the RetS transmitter domain of *A. vinelandii* also indicates that it could not have kinase activity. In *A. vinelandii*, the phenotypes of the *retS* mutation strongly suggest that the RetS-GacS interaction did not promote the activation of GacS and therefore the activation of GacA.

One possible explanation for the phenotypes of the *retS* mutant in *A. vinelandii* could be the potential inhibition of the phosphatase activity of GacS by dimerization with RetS. In *E. coli*, UvrY, the GacA homolog, is sometimes phosphorylated by acetyl phosphate, independent of BarA (GacS homolog) [50]. BarA has both kinase and phosphatase activity and, under specific conditions, dephosphorylates UvrY by acting as a homodimer. If a similar process occurs in the GacS/A system of *A. vinelandii*, RetS interference with the ability of GacS to form homodimers could reduce the phosphatase activity of GacS, favoring the phosphorylated state of GacA. Therefore, under specific conditions, the absence of RetS shifts the kinase/phosphatase balance of GacS toward phosphatase activity, deactivating GacA and negatively impacting its regulatory targets.

HptB can interact with RetS; in this study, we prove that this interaction also occurred in *A. vinelandii*. In *P. aeruginosa*, HptB can phosphorylate RetS; however, how this phosphorylation impacts RetS function is unclear. In *A. vinelandii*, HptB likely transfers its phosphate to RetS; in turn, phosphorylated RetS could transfer the phosphate to GacS through a similar mechanism to that described for the phosphorylation of GacS by LadS [8]. If this occurred in *A. vinelandii*, the positive regulatory phenotype of RetS could be explained. In this sense, determining the role that the two REC domains could play in the transfer of the phosphate group by HptB would be interesting.

The verification of the proposed hypotheses would be a very interesting subject of study for subsequent studies, which would improve the knowledge of the MKN-GacS. Although the presence of MKN-GacS is presumed in numerous species of the genus *Pseudomonas*, it has been studied in only a few species. The MKN of *P. aeruginosa* has become the study paradigm of this signaling system; the MKNs of *P. protegens* and *P. syringae*, although they have been less studied, show conserved aspects and other variables that suggest that the network is flexible and could have unique features in each bacterium. Outside of the *Pseudomonas* genus, the MKN has been studied only in *A. vinelandii*, where interesting variants have been found; there is no homolog for *ladS*, and a hybrid kinase (HrgS) related to GacS has not been reported in *Pseudomonas* species [14]. The positive regulation of GacS/A-related phenotypes by RetS shown in this study is another unique feature of the system that opens a new line of investigation.

## Conclusion

In *A. vinelandii*, RetS positively regulates the synthesis of alginates, PHB, and ARs and motility.

## Supplementary Information

Below is the link to the electronic supplementary material.

Supplementary file1 (DOCX 20 KB)

Fig. S1 Predicted domains of histidine kinase RetS homologs from *A. vinelandii * and *P. aeruginosa*. The analysis was carried out using the SMART protein domain annotation resource (http://smart.embl-heidelberg.de/) (DOCX 1224 KB)

Fig. S2 RT-PCR analysis of *retS* and *hsp70* genes in *A. vinelandii* E*retS* mutants. A) Genetic map of *retS*::Km and hsp70 in *A. vinelandii retS* mutants. Arrows indicate the *orf*s, an inverted triangle shows the insertion of the resistance cassette, the primer PCR pairs are indicated, and the amplicons generated are shown as squares. B) Agarose gel electrophoresis of RetS RT-PCR products of lanes: 1) GeneRuler 100bp DNA Ladder (Thermo-Scientific), 2) Strain E (WT), 3) E*ret*SP, 4) E*ret*SNP, 5) Negative control (PCR without template), 6) Positive control (PCR using genomic DNA as template). C) Agarose gel electrophoresis of Hsp70 RT-PCR products of lanes: 1) GeneRuler 100bp DNA Ladder (Thermo Scientific), 2) Strain E (WT), 3) E*ret*SP, 4) E*ret*SNP, 5) Negative control (PCR without template), 6) Positive control (PCR using genomic DNA as template) (DOCX 1160 KB)

Fig. S3 Alignment of the *A. vinelandii* and *P. aeruginosa* GacS proteins. Predicted domains are indicated by boxes: blue box, HAMP domain; black box, Transmitter domain (H1); red box, DHp subdomain; green box, receiver domain (D1); orange box, Hpt domain (H2) (DOCX 1325 KB)

Fig. S4 Alignment of the *A. vinelandii* and *P. aeruginosa* RetS proteins. The predicted transmitter domain (H1) is indicated by the black box (DOCX 1544 KB)
