# Supplementary material for: A Homolog of the Histidine Kinase RetS Controls the Synthesis of Alginates, PHB, Alkylresorcinols, and Motility in Azotobacter vinelandii
Source: Curr Microbiol. 2024 Aug 17;81(10):311. doi: 10.1007/s00284-024-03835-1 (PMC11330419; doi:10.1007/s00284-024-03835-1)
Supplement: Supplementary file 1 — Supplementary file1 (DOCX 20 KB) [file 284_2024_3835_MOESM1_ESM.docx]

**Table S1 Bacterial strains, plasmids and oligonucleotides used in this work.**

| Strain, plasmid or oligonucleotide | Relevant characteristics | Source or Reference |
| --- | --- | --- |
| ***A. vinelandii* strains** |  |  |
| E | mucoid, wild-type strain, also named AEIV | 30 |
| DJ | *algU*, non-mucoid strain | 29 |
| E*gacS* | E derivate with a *gacS* mutation | This work |
| E*retS*P | E derivate with a *retS* polar mutation | This work |
| EretSNP | E derivate with a *retS* non-polar mutation | This work |
| E*Avin_retS*P/  *melA::retS* | E*Avin retS*P complemented in cis which carried wild-type *retS* recombined in *melA* locus | This work |
| E*Avin_retSgacS* | E derivative with a *gacS* and *Avin_34990* mutations | This work |
| EP*rsmZ1*-*gusA* | E strain derivative, carrying a P*rsmZ1*-*gusA* transcriptional fusion | 24 |
| EP*rsmZ2*-*gusA* | E strain derivative, carrying a P*rsmZ2*-*gusA* transcriptional fusion | 24 |
| Ep*rsmZ6*-*gusA* | E strain derivative, carrying a P*rsmZ6*-*gusA* transcriptional fusion | 24 |
| EP*rsmY*-*gusA* | E strain with a P*rsmY*-*gusA* transcriptional fusion recombined into *melA* locus | 25 |
| EP*rsmZ1*-*gusAretS* | EP*rsmZ1-gusA* with a *retS* mutation | This work |
| EP*rsmZ2*-*gusAretS* | EP*rsmZ2-gusA* with a *retS* mutation | This work |
| EP*rsmZ6*-*gusAretS* | EP*rsmZ6-gusA* with a *retS* mutation | This work |
| EP*rsmY*-*gusAretS* | EP*rsmY-gusA* with a *retS* mutation | This work |
| ***E. coli* strains** |  |  |
| DH5α | F^-^ Φ80*lac*Z∆M15 ∆ (*lac*ZYA-*argF*) U169 *recA1endA1 hsd*R17(r_k_^-^, m_k_^+^) *phoAsup*E44 *thi*-1 *gyr*A96 *rel*A1 λ^-^ | Invitrogen |
| SU202 | *lexA*71 ::Tn 5(Def)*sulA*211 Δ (*laclPOZYA*) 169/F/*laclqlacZ*ΔM 15::Tn9 | 36 |
|  |  |  |
| **Plasmids** |  |  |
| pGEM-T Easy | PCR cloning vector | Promega |
| pSUP202 | Conjugative cloning vector | 34 |
| pGEM*retS*1.5 | pGEMT-Easy carrying a 1.581-kb fragment containing *retS* locus | This work |
| pGEM*retS*wt | pGEMT-Easy carrying a 1.5-kb fragment containing *retS* *orf* plus its regulatory region. | This work |
| pGEM*retS*::KmNP | pGEM*retS*1.5 carrying a ret*S*::Km non-polar mutation | This work |
| pGEM*retS*::KmP | pGEM*retS*1.5 carrying a ret*S*::Km polar mutation | This work |
| pUMATc | Integrative cloning vector, ampicillin and tetracycline resistant | 35 |
| pMC7 | pBluescript II KS carrying a *gacS*::Sm/Sp non-polar mutation | 33 |
| pSUP*gacS*::Sm | pSUP202 carrying a *gacS*::Sm/Sp non-polar mutation | This work |
| pSR658 | ColE1 expression plasmid for LexA dimerization system; Tc^r^. | 36 |
| pSR659 | p15A expression plasmid for LexA dimerization system; Amp^r^. | 36 |
| pSR658RetS | pSR558 carrying a fragment of that encoded the cytoplasmic part of RetS | 14 |
| pSR659GacS | pSR559 carrying a fragment of that encoded the cytoplasmic part of GacS | 14 |
| pSR659HptB | pSR559 carrying the complete *hptB* gene | This work |
| pBSL98 | Source of the Km resistance cassette | 31 |
| **Oligonucleotides (5'-3')** | | |
| FRetSZ-BH1 | GGA TTC GCC AAG AGC GAA TTC CTG | This work |
| RRetS-S1 | TAT GTC GAC TCA GGG ATG AAG CGC GTG | This work |
| WTS2D | TCC CTG CGC AGC CAC GAA CT | 14 |
| WTS2R | GGC GCC GAA TTT CGC CTC G | 14 |
| ER1RR_RetS_Fw | ATGAGAATCCCTGAAAGCCTGCGATAATCA | This work |
| Hsp70_RTIRv | CGC TCC TCG AAG TTG AAG AA | This work |
| hptBTHFw | GAGCTCGTGTCCGAACATCTCGAT | This work |
| hptBTHRv | GGTACCTCACGGATGACGCTGTCG | This work |
