# Supplementary material for: A Homolog of the Histidine Kinase RetS Controls the Synthesis of Alginates, PHB, Alkylresorcinols, and Motility in Azotobacter vinelandii
Source: Curr Microbiol. 2024 Aug 17;81(10):311. doi: 10.1007/s00284-024-03835-1 (PMC11330419; doi:10.1007/s00284-024-03835-1)
Supplement: Supplementary file 2 — Fig. S1 Predicted domains of histidine kinase RetS homologs from A. vinelandii and P. aeruginosa. The analysis was carried out using the SMART protein domain annotation resource (http://smart.embl-heidelberg.de/) (DOCX 1224 KB) [file 284_2024_3835_MOESM2_ESM.docx]

Transmitter (H1)

Rec (D)

Rec (D)

Input (7TMR-DISMED2)

RetS *A. vinelandii*

RetS *P. aeruginosa*

Fig. S1
