# Supplementary figures and images for: A Homolog of the Histidine Kinase RetS Controls the Synthesis of Alginates, PHB, Alkylresorcinols, and Motility in Azotobacter vinelandii
Source: Curr Microbiol. 2024 Aug 17;81(10):311. doi: 10.1007/s00284-024-03835-1 (PMC11330419; doi:10.1007/s00284-024-03835-1)

hsp70

*retS*

Fig. S2

*hsp70*


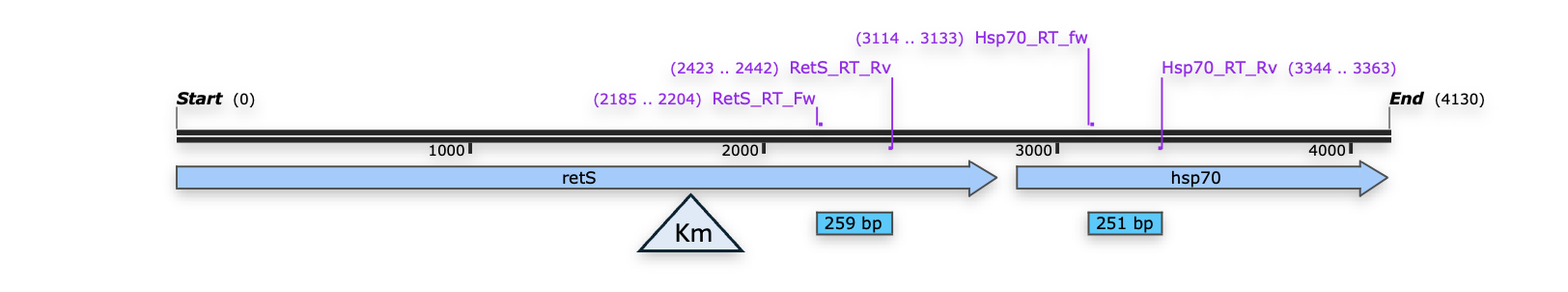


RT-PCR

Amplicon

*retS*

RT-PCR

Amplicon

*hsp70*

1

2

3

4

5

6


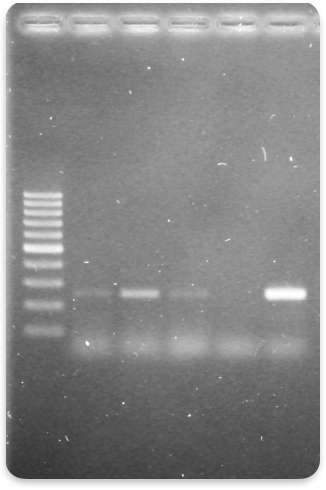


1

2

3

4

5

6


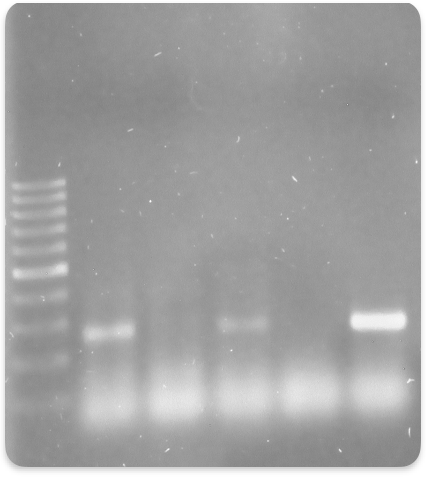


A

B

C

Supplement: Supplementary file 3 — Fig. S2 RT-PCR analysis of retS and hsp70 genes in A. vinelandii EretS mutants. A) Genetic map of retS::Km and hsp70 in A. vinelandii retS mutants. Arrows indicate the orfs, an inverted triangle shows the insertion of the resistance cassette, the primer PCR pairs are indicated, and the amplicons generated are shown as squares. B) Agarose gel electrophoresis of RetS RT-PCR products of lanes: 1) GeneRuler 100bp DNA Ladder (Thermo-Scientific), 2) Strain E (WT), 3) EretSP, 4) EretSNP, 5) Negative control (PCR without template), 6) Positive control (PCR using genomic DNA as template). C) Agarose gel electrophoresis of Hsp70 RT-PCR products of lanes: 1) GeneRuler 100bp DNA Ladder (Thermo Scientific), 2) Strain E (WT), 3) EretSP, 4) EretSNP, 5) Negative control (PCR without template), 6) Positive control (PCR using genomic DNA as template) (DOCX 1160 KB) [file 284_2024_3835_MOESM3_ESM.docx]

Fig. S3


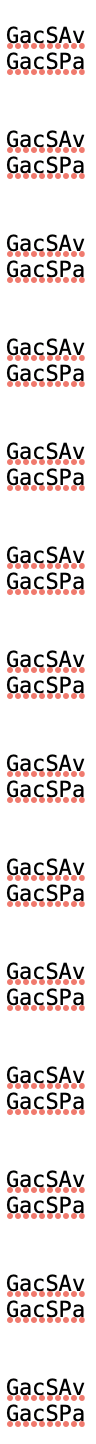

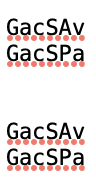

Supplement: Supplementary file 4 — Fig. S3 Alignment of the A. vinelandii and P. aeruginosa GacS proteins. Predicted domains are indicated by boxes: blue box, HAMP domain; black box, Transmitter domain (H1); red box, DHp subdomain; green box, receiver domain (D1); orange box, Hpt domain (H2) (DOCX 1325 KB) [file 284_2024_3835_MOESM4_ESM.docx]
