# Supplementary material for: A Homolog of the Histidine Kinase RetS Controls the Synthesis of Alginates, PHB, Alkylresorcinols, and Motility in Azotobacter vinelandii
Source: Curr Microbiol. 2024 Aug 17;81(10):311. doi: 10.1007/s00284-024-03835-1 (PMC11330419; doi:10.1007/s00284-024-03835-1)
Supplement: Supplementary file 4 [file 284_2024_3835_MOESM5_ESM.docx]

```
RetSAv      MRRPWNATTLIVSLLLVVLAALP-TLAGSLLR-SDEQFGPLGPGAPNGDWRILLDESASL      58
RetSPa      MVRLRIAIGLLVSFLLLLLGPMSPAVADDAGVSSVPLQTTATTPSANQNWRLLRDESAQL      60
            * *   *  *:**:**::*. :  ::*..    *          : * :**:* ****.*

RetSAv      TLKDVIERRDHFAPLGHRSLTLPAN-QAAWLRVSIAGHDTPRWIWVFAPRVDRVDFFLTN      117
RetSPa      RIADVLQRKEQFRPLAKRSFIFPASPQAVWLQVQLPAQKVPSWLWIFAPRVQYLDYYLVQ      120
             : **::*:::* **.:**: :**. **.**:*.: .:..* *:*:*****: :*::*.:

RetSAv      RGATERRIETGAMLPDG-LSTSGQAHLFDLPTDQTTREVWLRLAPRQAAPAWFDYVDTAG      176
RetSPa      DGQLVRDQHTGESRPFQERPLPSRSYLFSLPVDGKPMTLYVRMTSNHPLMAWFDQIDEAG      180
             *   *  .**   *       .:::**.**.* .   :::*:: .:    **** :* **

RetSAv      LLDKNKLAYTLGALLSALALGMIYHLVRFGYNRVLCNLWLSAMQGTLLLSAIAHFGLLGA      236
RetSPa      LVGLEKPAYAFGMLLGGMLLLLMYNLIRFAYSRSASSLWLAAVHAALAVCAAANLGLVAF      240
            *:. :* **::* **..: * ::*:*:**.*.*  ..***:*::.:* :.* *::**:.

RetSAv      WLPQLGHYQIRVADITALLSFLFLLQFTRSFFDQEESPRLLDWLLRGEIAVLALAAVLDG      296
RetSPa      WLPGLKFNQSLTADLGALGAAVSLLWFACSFFRGTAESRLNRLLQGEALLILAVGAIIAF      300
            *** * . *  .**: ** : : ** *: ***     . **   *    : :**:.*::

RetSAv      AS--PHDMLRSGLILLASLSATGCALYHWLHGYRPARLALVGTLLFHLGFSAYLSMLLDF      354
RetSPa      TQQLWFSWLIYLLVILSSLSVPLIAAWHWYRGYQPARLIVAGMIVFNAGFMVFLPVLFGT      360
            :.    .. *   *::*:***.    * :** :**:**** :.* ::*: ** .:* :*:.

RetSAv      GQFGPCWQIFALFAFTAASGLLLVFALAERQCKIERDRAAWRTSEAASSAELRAKSEFLA      414
RetSPa      KQLDPGWLVLGVFSFATLAGLVLSVSLTERQRLIQQLNLQQRTSEAAHTAELQTKAEFLA      420
             *:.* * ::.:*:*:: :**:* .:*:*** *:: .    ****** :***::*:****

RetSAv      KLSHEIRTPMNGVLGMTELLLGTPLTAKQYDYVQTINSSGNELLSLINDILDISRLESGQ      474
RetSPa      KISHEIRTPMNGVLGMTELLLGTPLSAKQRDYVQTIHSAGNELLTLINEILDISKLESGQ      480
            *:***********************:*** ******:*:*****:***:*****:*****

RetSAv      IELDDVQFDLNGLIEDCLGIFRAKAERRHVELISFIQPQVPRVINGDPTRLRQILLTLIE      534
RetSPa      IELDEVQFDLNALIEDCLDIFRVKAEQQRIELISFTQPQVPRVIGGDPTRLRQVVLSLLD      540
            ****:******.******.***.***::::***** ********.********::*:*::

RetSAv      NAFRQTDEGEILLKVSLETEGPRPNLHVAVQDSGEPMPESRREAMLNSRVQSSDLLSAAQ      594
RetSPa      NAFKQTEEGEILLVVALDDQGETPRLRIAVQDSGHPFDAKEREALLTAELHSGDFLSASK      600
            ***:**:****** *:*: :*  *.*::******.*:  ..***:*.:.::*.*:***::

RetSAv      IDGSLDLIIARQLITLMHGICGIDADPTRGTTLWLSLPLDPARLEHPVADLDSPLQDARL      654
RetSPa      LGSHLGLIIARQLVRLMGGEFGIQSGSSQGTTLSLTLPLDPQQLENPTADLDGPLQGARL      660
            :..  *.*******: ** *  **::. ::**** *:***** :**:*.****.***.***

RetSAv      LVVDDNDTCRKVLLQQCGAWGMKVSAVSSGKEALALLRTKADLGEYFDAALLDQNMPGMS      714
RetSPa      LVVDDNETCRKVLVQQCSGWGLNVSAVSSGKEALAQLRTKAHLREYFDVVLLDQDMPGMT      720
            ******:******:***..**::************ *****.* ****..****:****:

RetSAv      GLQLAQRIKEDPGLNHDILLVMLTGLSDAPGRVYARNAGITCSLAKPVAGYTLKTTLAEE      774
RetSPa      GMQLAAKIKEDPNLNHDILLIMLTGISNAPSKIIARNAGIKRILAKPVAGYTLKATLADE      780
            *:*** :***** *******:****:*:** :: ******    ***********:***:*

RetSAv      LGKRRAARPA--ASGLEENAPLEVPSDFRILVAEDNSISTKVIRGMLRKLNLQPDTASNG      832
RetSPa      LAQRGVSGVTNYLQPAKEAQAPSLPSDFRILVAEDNSISTKVIRGMLNKLNLQPDTASNG      840
            *.:* .:  :   .  :*     .:***********************.************

RetSAv      EEALQAMKAKQYDLVLMDCEMPVLDGFAATEQLRAWEASELRPHTPIVALTAHILSEHRE      892
RetSPa      QEALSAMKATQYDLVLMDCEMPVLDGFSATEQLRAWEAHEQRPHTPVVALTAHILSEHKE      900
            :***.****.*****************:********** * *****:***********:*

RetSAv      HAQRVGMDGHLAKPVELSQLRALIEHWVERKEESYRHALHP-  933
RetSPa      RARLVGMDGHMAKPVELSQLRELIAYWVGERDRRRQGDALPS  942
            :*: ******:********** ** :** .::.  :    *
```

Fig. S4
